# Maternal Outcomes and Resource Utilization in Pregnancy-Related Hospitalizations Among Patients With Irritable Bowel Syndrome: A Nationwide Inpatient Analysis, 2020-2022

**DOI:** 10.7759/cureus.101675

**Published:** 2026-01-16

**Authors:** Muhammad Haris Latif, Ayesha Kang, Emaan Mazhar, Kahee A Mohammed, Joel Riley, Hani El-Halawani, Kamran Qureshi

**Affiliations:** 1 Internal Medicine, SSM Health St. Mary's Hospital, St. Louis, USA; 2 Internal Medicine, Nishtar Medical University, Multan, PAK; 3 Gastroenterology, SSM Health St. Mary's Hospital, St. Louis, USA; 4 Gastroenterology, SSM Health Depaul Hospital, St. Louis, USA; 5 Hepatology, Saint Louis University School of Medicine, St. Louis, USA

**Keywords:** high-risk pregnancy, ibs, management of ibs, obstretic, pregnancy

## Abstract

Background

Irritable bowel syndrome (IBS) is common among women of reproductive age, yet its implications for pregnancy-related hospitalizations and maternal outcomes in contemporary inpatient settings remain incompletely characterized. Accordingly, this study aimed to address this gap.

Methods

We performed a retrospective, cross-sectional analysis of delivery-related hospitalizations from 2020 to 2022 using the Nationwide Inpatient Sample (NIS). IBS was identified using the International Classification of Diseases, Tenth Revision, Clinical Modification (ICD-10-CM) diagnosis codes documented during the delivery hospitalization. Survey-weighted analyses were used to generate nationally representative estimates. Multivariable regression models, adjusted for demographic factors, obstetric characteristics, medical comorbidities, psychiatric conditions, and substance use, were used to evaluate the associations between IBS and maternal outcomes, length of stay, and total hospital charges. Sensitivity analyses examined placenta-specific outcomes and postpartum infections using more granular outcome definitions.

Results

Among 2,086,607 weighted pregnancy-related hospitalizations, 4,889 (0.23%) had a documented diagnosis of IBS. Patients with IBS were older and had a higher burden of psychiatric, metabolic, and substance-related comorbidities. After multivariable adjustment, IBS was independently associated with prolonged length of stay (adjusted odds ratio (aOR): 1.29, 95% confidence interval (CI): 1.13-1.48), higher hospital charges (adjusted mean increase: $1,656, 95% CI: $769-$2,544), placental complications (aOR: 1.23, 95% CI: 1.08-1.40), and postpartum infection (aOR: 1.41, 95% CI: 1.00-1.98). IBS was not associated with major maternal complications or mortality. Sensitivity analyses demonstrated outcome specificity.

Conclusions

In this contemporary nationwide inpatient analysis, IBS was infrequently documented during pregnancy-related hospitalizations but was associated with an increased comorbidity burden, resource utilization, and select obstetric complications. These findings suggest that IBS in pregnancy functions as a marker of obstetric complexity rather than a strong independent risk factor for most major maternal complications.

## Introduction

Irritable bowel syndrome (IBS) is a chronic disorder of gut-brain interaction characterized by abdominal pain and altered bowel habits, and it disproportionately affects women during their reproductive years [1,2,3]. Pregnancy represents a unique physiological state characterized by profound hormonal, immunologic, and autonomic changes that influence gastrointestinal motility, visceral sensitivity, and stress responsiveness [4,5,6]. These pregnancy-related changes may modify the expression of IBS symptoms, complicate diagnostic recognition, and potentially alter the relationship between IBS and obstetric outcomes [5]. Despite the high prevalence of IBS among women of childbearing age, its implications during pregnancy, particularly in hospitalized populations, remain not fully understood.

Population-based studies have suggested that IBS may be associated with adverse reproductive outcomes earlier in pregnancy, including miscarriage and ectopic pregnancy [7,8,9]. In a large contemporary claims-based analysis, the prevalence of IBS among pregnant and postpartum individuals was reported to be 1.38 percent. Small prospective and observational studies have demonstrated that functional bowel symptoms are common during pregnancy and are associated with impaired quality of life, increased psychological distress, and greater healthcare utilization [5,6]. However, symptom-based studies are limited in their ability to evaluate infrequent yet clinically meaningful maternal outcomes. From a biological standpoint, several mechanisms support a potential association between IBS and pregnancy-related outcomes, including dysregulation of the gut-brain axis, altered stress responsiveness, immune modulation, and autonomic nervous system changes that occur during gestation [2,6]. In addition, IBS has been associated with variations in healthcare engagement and diagnostic intensity, which may influence inpatient recognition and management during pregnancy.

Most data examining later pregnancy outcomes come from large administrative datasets. Prior analyses using the Nationwide Inpatient Sample (NIS) during the International Classification of Diseases, Ninth Revision (ICD-9) era demonstrated that IBS-coded delivery hospitalizations were associated with higher odds of several maternal and neonatal complications, including hypertensive disorders, venous thromboembolism, gestational diabetes, and congenital anomalies [8]. These studies also consistently reported that pregnant patients with IBS differed demographically from those without IBS, tending to be older, more frequently White, and more likely to carry psychiatric, metabolic, and endocrine comorbidities [10,11,12]. While informative, these analyses were conducted in earlier eras of obstetric practice and coding and did not fully evaluate hospital resource utilization or a broad range of maternal complications.

More recent claims-based studies in the ICD-10 era have reinforced the observation that IBS in pregnancy is strongly associated with psychiatric comorbidity and multimorbidity [12]. These findings underscore the importance of distinguishing whether adverse outcomes attributed to IBS reflect the condition itself or the complex clinical context in which IBS occurs. At the same time, contemporary data on inpatient outcomes, including length of stay, hospital charges, placental pathology, and postpartum infectious complications, remain sparse.

Several key gaps, therefore, remain. First, it is unclear whether associations reported in earlier NIS studies persist in more recent years, particularly after comprehensive adjustment for psychiatric, metabolic, and substance-related comorbidities. Second, the relationship between IBS and hospital resource utilization during pregnancy has not been well characterized at the national level. Third, outcomes reflecting obstetric complexity, including placental complications and postpartum infections, have received limited attention despite biologic plausibility and potential clinical relevance. Finally, rare maternal outcomes require very large, nationally representative samples and rigorous analytic approaches to address event scarcity and model instability.

The objectives of this study were (1) to characterize the demographic and comorbidity profile of pregnant patients with IBS hospitalized for delivery in the United States; (2) to evaluate associations between IBS and maternal, postpartum, and placental complications using survey-weighted multivariable models; and (3) to assess inpatient resource utilization, including length of stay and hospitalization charges. Prespecified sensitivity analyses were performed to evaluate the specificity of observed associations with postpartum infectious outcomes.

## Materials and methods

Data source

We conducted a retrospective, cross-sectional study using the NIS, a publicly available, all-payer database developed by the Healthcare Cost and Utilization Project (HCUP). The NIS approximates a 20% stratified sample of U.S. hospital discharges and provides discharge-level sampling weights that allow for nationally representative estimates [13,14,15,16,17]. Reported hospital charges reflect total billed charges for the hospitalization and do not represent direct patient out-of-pocket costs or actual reimbursements. Because patient financial responsibility varies by insurance coverage and negotiated payment structures, primary payer status was included as a covariate in all multivariable analyses assessing resource utilization. We analyzed delivery-associated hospitalizations from 2020 to 2022. Each study year represents an independent, nationally representative sample. The dataset includes patient demographics, diagnoses, procedures, hospital characteristics, length of stay, and total charges. Because the NIS contains deidentified data, this study was exempt from institutional review board oversight.

Study population

Analyses were restricted to delivery-related hospitalizations. Pregnancy losses, including miscarriage and ectopic pregnancy, were not evaluated. As the NIS is a discharge-level database that lacks patient identifiers, longitudinal follow-up across years was not possible; therefore, analyses were conducted at the hospitalization level.

Exposure definition

The primary exposure of interest was IBS, identified using the International Classification of Diseases, Tenth Revision, Clinical Modification (ICD-10-CM) diagnosis codes (K58.x) documented during the delivery hospitalization [4,13]. IBS was analyzed as a binary variable (present vs. absent). Because IBS is predominantly managed in the outpatient setting, its identification in the NIS reflects inpatient documentation and is expected to underestimate true population prevalence [13,14].

Outcomes

Maternal outcomes included cesarean delivery, gestational diabetes, hypertensive disorders of pregnancy, venous thromboembolism, and placental complications. Placental complications were examined both as a composite outcome and as individual conditions, including placenta previa, placental abruption, and placenta accreta spectrum disorders. Postpartum outcomes included postpartum infection, postpartum hemorrhage, thromboembolic events, and mood disorders. Length of stay was dichotomized as prolonged versus non-prolonged based on established NIS conventions.

Covariates

Baseline maternal demographic characteristics included age, race/ethnicity, and primary payer. Medical and behavioral comorbidities were selected a priori based on clinical relevance and prior literature. Obstetric characteristics included multiple gestation, cesarean delivery, and placental complications. Medical comorbidities included diabetes mellitus, hypertension, obesity, severe obesity, dyslipidemia, chronic kidney disease, and thyroid disorders. Psychiatric and substance-related conditions included depression, anxiety disorders, post-traumatic stress disorder, bipolar disorder, psychotic disorders, tobacco use, alcohol use disorder, opioid use disorder, cannabis use, and cocaine use. Variables with extremely low prevalence or evidence of perfect prediction were not included in multivariable models to maintain model stability. Baseline comorbidities were compared between IBS and non-IBS pregnancies using survey-weighted tabulations, with results reported as column percentages.

Statistical analysis

All analyses accounted for the complex survey design of the NIS, incorporating discharge-level sampling weights, stratification, and clustering to generate nationally representative estimates [15,18]. Continuous variables were summarized using weighted means, and categorical variables were summarized using weighted proportions. Comparisons between groups were assessed using design-based statistical methods. Multivariable survey-weighted logistic regression models were used to estimate adjusted odds ratios (aORs) and 95% confidence intervals (CIs) for the association between IBS and each outcome. All models adjusted for maternal age, race/ethnicity, primary payer, diabetes mellitus, chronic hypertension, obesity, dyslipidemia, smoking, substance use, anxiety, depression, chronic kidney disease, and congestive heart failure.

Due to the low prevalence of IBS and the rarity of certain outcomes, models with evidence of perfect prediction or insufficient events were interpreted cautiously, and estimates were reported as imprecise when applicable [19,20]. Statistical significance was defined as a two-sided p-value <0.05. All analyses were performed using Stata with survey (svy) commands. To minimize overadjustment bias, pregnancy complications were not included as covariates in models where they served as the outcome of interest [21,22,23]. For postpartum outcomes, models additionally adjusted for major pregnancy complications when clinically appropriate. Survey-weighted linear regression was used to assess associations with total hospital charges. Multivariable models adjusted for demographics, obstetric factors, medical comorbidities, psychiatric conditions, and substance use.

Sensitivity analyses

Several sensitivity analyses were predefined to assess the specificity of observed associations. First, the composite placental complications outcome was disaggregated into individual diagnoses, including placenta previa, placental abruption, and placenta accreta spectrum disorders. Separate survey-weighted logistic regression models were run for each placental outcome using the same covariate structure as the primary models.

Second, to evaluate whether the association between IBS and postpartum infection was confounded by placental pathology, an additional model was constructed for postpartum infection with further adjustment for placental complications. This analysis was designed to determine whether IBS remained associated with postpartum infection independently of placental disease.

Third, postpartum endometritis was examined as a distinct outcome to evaluate whether the observed association with postpartum infection was driven by severe uterine infection. These models included additional adjustments for cesarean delivery and placental complications. Because endometritis was a rare outcome, some covariates were omitted due to perfect prediction, and the results were interpreted cautiously because of potential model instability.

Finally, for outcomes with very low event rates, including maternal mortality, placenta accreta spectrum disorders, and embolic events, model diagnostics were reviewed for evidence of rare-event bias, wide confidence intervals, and separation. In these cases, null findings were interpreted as reflecting limited statistical power rather than definitive evidence of no association. Across all sensitivity analyses, consistency in the direction and magnitude of associations was emphasized over isolated statistical significance.

Ethics and data statement

This study utilized de-identified, publicly available data from the NIS, part of the HCUP maintained by the Agency for Healthcare Research and Quality (AHRQ). Because the NIS contains no direct patient identifiers and complies with the Health Insurance Portability and Accountability Act (HIPAA) privacy standards, this analysis was considered exempt from institutional review board (IRB) oversight under 45 CFR 46.104(d)(4). Therefore, informed consent was not required.

## Results

Study population and IBS prevalence

A total of 2,086,607 weighted pregnancy-related hospitalizations from 2020 to 2022 were identified. (Table 1). Among these, 4,889 admissions (0.23%) had a documented diagnosis of IBS, corresponding to a survey-weighted prevalence of 0.23% (95% CI: 0.23-0.24%).

**Table 1:** Survey-weighted prevalence of IBS among pregnancy-related hospitalizations in the United States (NIS 2020–2022) IBS: irritable bowel syndrome; NIS: Nationwide Inpatient Sample; CI: confidence interval

Year	Total delivery-related hospitalizations	IBS cases (n)	Prevalence (%)	Prevalence per 100,000 (95% CI)
2020	690,751	1,485	0.21	215 (204–226)
2021	697,521	1,745	0.25	250 (238–262)
2022	698,335	1,659	0.24	238 (226–249)
Overall	2,086,607	4,889	0.23	234 (228–241)

Baseline demographic characteristics

Pregnant patients with IBS were older than those without IBS (mean age: 30.8 vs. 29.4 years, p<0.001). IBS prevalence differed significantly by race and payor status (Table 2). IBS was most frequently observed among White patients (0.36%) and among privately insured admissions (0.32%), while prevalence was lower among African American and Hispanic patients and among Medicaid-insured hospitalizations (design-based p<0.001 for both comparisons).

**Table 2:** Baseline demographic characteristics of pregnancy-related hospitalizations by IBS status (NIS 2020–2022) IBS: irritable bowel syndrome; NIS: Nationwide Inpatient Sample; SE: standard error; CI: confidence interval; LOS: length of stay; aOR: adjusted odds ratio

Characteristic	Non IBS	IBS	P-value
Weighted admissions, n (%)	2,081,718 (99.77)	4,889 (0.23)	
Age, mean (SE), by year	29.37 (0.004)	30.80 (0.07)	<0.001
	Adjusted effect	95% CI	P-value
Prolonged LOS	aOR 1.29	1.13–1.48	<0.001
Total charges (absolute)	$1,656	$769–$2,544	<0.001
Race/ethnicity, %		% among IBS	<0.001
White		0.36	
Black		0.12	
Hispanic		0.09	
Asian/Pacific Islander		0.08	
Native American		0.13	
Other		0.14	
Primary payor, %			<0.001
Medicare		0.37	
Medicaid		0.13	
Private insurance		0.32	
Self-pay		0.11	
No charge		0.06	
Other		0.27	

Length of stay and resource utilization

After multivariable adjustment, IBS was independently associated with prolonged length of stay (aOR: 1.29, 95% CI 1.13-1.48) and higher hospitalization charges (adjusted mean difference: $1,656, 95% CI: $769-$2,544). These findings remained significant after accounting for payor status, obstetric complexity, medical comorbidities, psychiatric conditions, and substance use.

Comorbidity profile

Psychiatric comorbidities were disproportionately prevalent among IBS hospitalizations, particularly anxiety and depression, alongside higher rates of metabolic and substance-related conditions (Table 3).

**Table 3:** Comorbidity burden among inpatient pregnant patients with and without IBS IBS: irritable bowel syndrome

Comorbidity	No IBS (%)	IBS (%)	P-value
Diabetes mellitus	0.89	0.78	0.400
Hypertension	1.23	1.66	0.006
Chronic kidney disease	0.12	0.25	0.010
Obesity	10.11	13.64	<0.001
Morbid obesity	3.8	5.05	<0.001
Dyslipidemia	0.45	2.17	<0.001
Smoking	10.78	15.89	<0.001
Substance use disorder	5.97	7.34	<0.001
Depression	5.63	21.39	<0.001
Anxiety disorder	8.21	37.21	<0.001

Maternal outcomes

IBS was not independently associated with cesarean delivery, preeclampsia, gestational diabetes mellitus, venous thromboembolism, postpartum hemorrhage, postpartum embolic events, postpartum mood disorders, or in-hospital maternal death. Analyses of mortality were limited by the rarity of events and perfect prediction. IBS was, however, independently associated with placental complications and postpartum infection, while no significant associations were observed for other major maternal or psychiatric outcomes (Table 4).

**Table 4:** Adjusted inpatient maternal outcomes associated with IBS in pregnancy IBS: irritable bowel syndrome; OR: odds ratio; CI: confidence interval

Maternal Outcome	Adjusted OR	95% CI	P-value
In-hospital mortality	-	-	-
Cesarean delivery	0.64	0.27–1.54	0.32
Preeclampsia	1.05	0.94–1.17	0.41
Gestational diabetes mellitus	0.97	0.88–1.07	0.50
Venous thromboembolism	0.89	0.22–3.57	0.87
Placental complications	1.23	1.08–1.40	0.003
Postpartum hemorrhage	1.05	0.93–1.19	0.45
Postpartum infection	1.41	1.00–1.98	0.048
Endometritis	1.37	0.34–5.45	0.66
Postpartum embolism	0.51	0.07–3.60	0.50
Postpartum mood disorder	0.92	0.47–1.77	0.80
Other postpartum complications	1.10	0.99–1.22	0.088

Sensitivity analyses

These findings further support the stability of the primary associations; placental complications were disaggregated into individual diagnoses. IBS was not associated with placenta previa or placental abruption and showed a non-significant trend toward placenta accreta spectrum disorders. The association between IBS and postpartum infection persisted after additional adjustment for placental complications (aOR: 1.40, 95% CI: 1.00-1.97, p=0.050). In contrast, IBS was not associated with postpartum endometritis after adjustment for placental complications and cesarean delivery (aOR: 1.37, 95% CI: 0.34-5.45, p=0.66) (Table 5), indicating that the observed infectious risk was not driven by severe uterine infection.

**Table 5:** Sensitivity analyses of inpatient placental and postpartum outcomes associated with IBS IBS: irritable bowel syndrome; OR: odds ratio; CI: confidence interval

Sensitivity outcome	Adjusted OR	95% CI	P-value
Placenta previa	1.08	0.80–1.45	0.61
Placental abruption	0.99	0.76–1.28	0.92
Placenta accreta spectrum	1.60	0.91–2.83	0.10
Postpartum infection (adjusted for placental complications)	1.40	1.00–1.97	0.05
Postpartum endometritis (adjusted for placental complications and cesarean delivery)	1.37	0.34–5.45	0.659

## Discussion

In this nationally representative analysis of pregnancy-related hospitalizations from 2020 to 2022, we found that IBS, although infrequently coded in inpatient obstetric admissions, was associated with a substantially higher comorbidity burden, prolonged length of stay, increased inpatient costs, and higher odds of placental complications and postpartum infection. In contrast, IBS was not independently associated with most major maternal complications, including hypertensive disorders of pregnancy, gestational diabetes mellitus, venous thromboembolism, cesarean delivery, or in-hospital mortality. Sensitivity analyses using more granular outcome definitions supported the specificity of these findings. Taken together, our results suggest that IBS in pregnancy functions primarily as a marker of obstetric complexity and healthcare utilization rather than a direct driver of severe maternal morbidity [14].

Comparison with prior literature

Our findings are consistent with and extend prior work examining IBS in pregnancy across multiple study designs. Population-based cohort studies have reported associations between IBS and adverse reproductive outcomes earlier in pregnancy, including miscarriage and ectopic pregnancy [8], supporting the concept that IBS may reflect broader systemic or psychosocial vulnerability during pregnancy rather than an isolated gastrointestinal condition.

The most directly comparable inpatient analysis is a prior NIS study conducted during the ICD-9 era, which reported that IBS was associated with higher odds of preeclampsia, venous thromboembolism, gestational diabetes, and congenital anomalies [8]. That study offered important early insights but differed substantially from the present analysis. It examined earlier periods of obstetric practice, relied on legacy coding frameworks, and included more limited adjustment for psychiatric and substance-related comorbidities. In contrast, our analysis reflects contemporary ICD-10-CM coding. It incorporates extensive adjustment for psychiatric, metabolic, and substance use conditions, factors that were highly prevalent among IBS hospitalizations in our cohort and likely confounded associations reported in earlier work.

More recent claims-based studies have reported substantially higher prevalence estimates of IBS among pregnant and postpartum populations than those observed in inpatient datasets. A large contemporary claims analysis reported an IBS prevalence exceeding 1% among pregnant and postpartum individuals and demonstrated strong associations with psychiatric comorbidities and multimorbidity [12]. These higher prevalence estimates likely reflect fundamental differences in case ascertainment. Claims-based studies capture outpatient and inpatient encounters longitudinally, whereas the NIS captures only diagnoses recorded during a single hospitalization [12,13]. Functional disorders such as IBS are commonly diagnosed and managed in outpatient settings and may not be documented during pregnancy-related admissions unless directly relevant to inpatient management. Consequently, the lower prevalence observed in our study reflects hospital-recognized IBS rather than true disease prevalence and should be interpreted accordingly.

Interpretation of maternal outcomes

After extensive adjustment, IBS was not independently associated with most major maternal complications, including hypertensive disorders, gestational diabetes, thromboembolism, or in-hospital maternal mortality. These null findings were consistent across multiple models and sensitivity analyses, suggesting that IBS alone does not confer a substantial independent risk for these outcomes, even when relevant comorbid conditions are accounted for. This contrasts with earlier NIS findings and likely reflects improved control for confounding, particularly for psychiatric and metabolic comorbidities that are now well recognized in IBS populations.

Notably, IBS was independently associated with placental complications and postpartum infections. While the underlying mechanisms remain uncertain, several biologically plausible pathways warrant consideration. IBS has been linked to altered stress responsivity, immune dysregulation, and gut-brain axis dysfunction, all of which may influence placental development and postpartum immune defense [24,25,26]. Sensitivity analyses demonstrated that the association with postpartum infection persisted after adjustment for placental complications, while IBS was not associated with postpartum endometritis when analyzed as a distinct outcome. These findings suggest that IBS may be associated with broader postpartum infectious vulnerability rather than severe uterine infection specifically.

Resource utilization and clinical complexity

One of the most consistent findings in our study was the association between IBS and greater inpatient resource utilization. Pregnant patients with IBS experienced longer hospital stays and higher total hospital charges, even after adjustment for demographic, obstetric, medical, psychiatric, and substance-related factors. These findings align with prior literature demonstrating increased healthcare utilization among patients with IBS in nonpregnant populations and extend this observation to hospitalized obstetric patients [8]. The high prevalence of psychiatric disorders, metabolic conditions, and substance use among IBS hospitalizations likely contributes to greater care complexity, diagnostic evaluation, and multidisciplinary involvement during pregnancy-related admissions. The observed increase in hospitalization charges among patients with IBS likely reflects increased clinical complexity, comorbidity burden, and obstetric risk, rather than direct patient financial responsibility. Importantly, these estimates represent hospital-level billed charges rather than patient out-of-pocket costs. Adjustment for insurance status reduces, but does not fully eliminate, institutional variation in billing practices.

Methodological considerations

Several aspects of our analytic approach enhance the validity of our findings. We used survey-weighted methods to generate nationally representative estimates and adjusted for a comprehensive set of prespecified covariates. Sensitivity analyses were performed to address outcome specificity and rare-event instability, including disaggregation of placental outcomes and separate modeling of postpartum endometritis, with additional adjustment for cesarean delivery and placental pathology. By emphasizing consistency of associations across models rather than isolated statistical significance, we sought to mitigate limitations inherent to rare outcomes in administrative data [27].

At the same time, our findings must be interpreted in the context of inpatient coding practices. The low observed prevalence of IBS likely reflects underdocumentation during pregnancy-related hospitalizations rather than true disease rarity. Accordingly, our results describe outcomes among patients with hospital-recognized IBS and should not be generalized to all pregnant individuals with IBS.

Clinical and research implications

Clinically, our findings suggest that IBS in pregnancy should be viewed as a marker of increased comorbidity burden and inpatient complexity, rather than a strong independent risk factor for most major maternal complications. Recognition of the high prevalence of psychiatric and metabolic comorbidities among pregnant patients with IBS may help inform risk stratification and care coordination [28,29]. From a research perspective, future studies that integrate outpatient, inpatient, and longitudinal data will be essential to better characterize the full spectrum of IBS in pregnancy and to clarify mechanistic pathways linking IBS to placental and infectious outcomes.

Limitations

This study has several important limitations inherent in the use of administrative inpatient data. First, IBS is a functional gastrointestinal disorder that is likely undercoded in the NIS. As a result, the observed prevalence reflects hospital-recognized IBS rather than true population prevalence [13,15]. Second, several maternal outcomes, particularly in-hospital mortality, endometritis, embolic events, and placenta accreta spectrum disorders, were rare, resulting in limited statistical power and episodes of model instability, including perfect prediction and variable omission. Accordingly, null associations for these outcomes should be interpreted with caution and should not be viewed as definitive evidence of no association. Third, the NIS lacks granular clinical detail, including symptom severity, outpatient disease course, medication use, and microbiome or inflammatory markers, which precludes mechanistic inference. Fourth, residual confounding related to healthcare utilization, diagnostic intensity [30], and unmeasured psychosocial factors may persist despite extensive multivariable adjustment. Finally, the cross-sectional study design limits causal inference, and observed associations should be interpreted as markers of obstetric complexity and resource utilization, rather than direct causal effects [30].

## Conclusions

In this contemporary nationwide analysis of pregnancy-related hospitalizations, IBS was infrequently documented during inpatient obstetric admissions but was consistently associated with greater comorbidity burden, increased resource utilization, and higher odds of select obstetric complications, including placental disorders and postpartum infections. In contrast, IBS was not independently associated with most major maternal complications or in-hospital maternal mortality after comprehensive adjustment for demographic, medical, psychiatric, and substance-related factors. These findings suggest that IBS in pregnancy primarily functions as a marker of clinical and psychosocial complexity, rather than a strong independent driver of severe maternal morbidity. Future studies that integrate longitudinal outpatient and inpatient data are needed to better characterize the full spectrum of IBS in pregnancy and to clarify mechanisms linking IBS to obstetric complexity and postpartum infectious risk.

## Appendices

**Table 6:** ICD-10-CM codes used for exposure, covariates, and outcomes ICD-10-CM diagnosis codes were identified from up to 40 discharge diagnosis fields (I10_DX1–I10_DX40). Composite outcomes were defined by the presence of ≥1 qualifying diagnosis code during the hospitalization. All analyses used discharge-level sampling weights to generate nationally representative estimates ICD-10-CM: International Classification of Diseases, Tenth Revision, Clinical Modification; IBS: irritable bowel syndrome; SGA: small-for-gestational age; NIS: Nationwide Inpatient Sample

Category	Variable	ICD-10-CM codes/definition
Exposure	IBS	K58.x
Obstetric characteristics	Multiple gestation	O30.x, O31.x
	Prior cesarean/uterine scar	O34.21, O34.219
	Cesarean delivery	ICD-10-PCS delivery procedure codes
Maternal medical comorbidities	Diabetes mellitus	E10.x–E14.x
	Hypertension	I10–I15
	Obesity	E66.x
	Morbid obesity	E66.01
	Dyslipidemia	E78.x
	Chronic kidney disease	N18.x
	Asthma	J45.x
	Hypothyroidism	E03.x
	Hyperthyroidism	E05.x
Psychiatric comorbidities	Anxiety disorders	F41.x
	Depressive disorders	F32.x, F33.x, F34.1
	Post-traumatic stress disorder	F43.1
	Bipolar disorder	F31.x
	Psychotic disorders	F20.x–F29.x
Substance use disorders	Tobacco use	F17.x, Z72.0
	Alcohol use disorder	F10.x
	Opioid use disorder	F11.x
	Cannabis use disorder	F12.x
	Cocaine use disorder	F14.x
	Other substance use	F13.x, F15.x, F16.x, F18.x, F19.x
Maternal outcomes	Gestational diabetes mellitus	O24.4x
	Gestational hypertension	O13.x
	Preeclampsia	O14.x
	HELLP syndrome	O14.2x
	Venous thromboembolism	O22.3x, O22.5x, O87.x
Placental complications	Placenta previa	O44.x
	Placental abruption	O45.x
	Placenta accreta spectrum	O43.2x
	Any placental complication (composite)	O44.x, O45.x, O43.2x
Postpartum outcomes	Postpartum hemorrhage	O72.x
	Postpartum infection (composite)	O85, O86.x
	Endometritis/puerperal sepsis (sensitivity analysis)	O85, O86.1
	Postpartum embolism	O88.x
	Postpartum mood disorders	F53.x
Neonatal outcomes	Preterm delivery	O60.x
	Stillbirth	O36.4x
	Fetal growth restriction/SGA	O36.59x, P05.x
	Neonatal respiratory distress	P22.x
	Neonatal sepsis	P36.x
Resource utilization	Prolonged length of stay	LOS above the year-specific 75th percentile
	Total hospital charges	NIS variable TOTCHG (billed charges)
